# Agreement Among Breast Cancer Screening Modalities in Breast Density Assessment and Cancer Risk Prediction

**DOI:** 10.1155/ijbc/4748963

**Published:** 2026-01-21

**Authors:** Areej S. Aloufi, Salman M. Albeshan, Abdulaziz S. Alshabibi, Meaad M. Almusined, Lina A. Aldibas, Milaf G. Alotaibi, Sara Hosawi

**Affiliations:** ^1^ Department of Radiological Sciences, College of Applied Medical Sciences, King Saud University, Riyadh, Saudi Arabia, ksu.edu.sa; ^2^ Department of Radiology, Breast Imaging, King Faisal Specialist Hospital and Research Center, Riyadh, Saudi Arabia, kfshrc.edu.sa

**Keywords:** breast cancer, DBT, dense breast, mammography, MRI, ultrasound

## Abstract

**Background:**

Breast density is increasingly recognized as a vital risk factor that affects early breast cancer detection. Therefore, this study was aimed at evaluating the agreement between different breast density measurements across multiple imaging modalities and identifying the best breast cancer predictor among these methods.

**Methods:**

Data for women over 30 years old who underwent mammography, synthetic mammography of digital breast tomosynthesis (SM‐DBT), ultrasound (US), and magnetic resonance imaging (MRI) were collected. Breast density was assessed by two breast radiologists using the American College of Radiology (ACR) categories and the visual analog scale (VAS) for percentage density (%PD). The agreement was calculated using the kappa coefficient (*k*) and Spearman correlation coefficient (*ρ*). Logistic regression odds ratios (ORs) were used to assess the best predictor of breast cancer based on breast density.

**Results:**

Among 77 women (mean age 47.34 years), 25 had breast cancer. Categorical breast density assessments showed the highest agreement between mammography and SM‐DBT (*k* = 0.535) and moderate agreement between mammography and MRI (*k* = 0.452). VAS analysis revealed moderate positive correlations between mammography and SM‐DBT (*ρ* = 0.49), mammography and MRI (*ρ* = 0.56), and SM‐DBT and MRI (*ρ* = 0.56), *p* < 0.05. Ultrasound showed the lowest correlation with all breast imaging modalities. Breast cancer risk prediction based on breast density showed significant associations for mammography (OR = 3.09) and MRI (OR = 4.16), *p* < 0.05.

**Conclusion:**

The results suggest notable variability in radiologists′ breast density assessment across different imaging modalities. MRI showed a greater ability to identify dense breast tissue and demonstrated potential value in breast cancer risk prediction, although these findings should be interpreted cautiously given the limited sample size. Establishing standardized approaches to breast density assessment remains important to improve the accuracy of breast cancer screening and risk prediction, and further research with larger cohorts is warranted.

## 1. Introduction

Breast density is increasingly recognized as a vital risk factor that affects early breast cancer detection. It is now well known that dense breast tissue increases the risk of developing breast cancer [1, 2]. In addition to the fact that it creates challenges when it comes to visualizing tumors due to the masking effect it represents in the mammography image [3], the United States has implemented the breast density notification law, which obligates informing women about these limitations and the possibility of other imaging modalities other than mammography in case of dense breast tissue [4]. The later‐stage diagnosis makes treatment for breast cancer even more complicated and uncertain [5]. This problem can be avoided by early detection through regular screening initiatives. In traditional radiology practice, mammography is the main method to assess breast density [6].

In general, mammography has been a key tool in screening for breast cancer since it provides an accurate and accessible method to detect early cases. However, recent studies, including large trials, have suggested greater benefit from adjunct or alternative imaging modalities like digital breast tomosynthesis (DBT), ultrasound (US), or magnetic resonance imaging (MRI) among women with higher breast densities [7]. Therefore, the European Society of Breast Imaging (EUSOBI) recommends using other breast imaging techniques besides mammography for females with denser breasts [7]. Therefore, it becomes even more necessary to ensure the reliability and consistency of breast density assessments across various imaging modalities. Disparities in assessing breast density may result in inconsistent risk estimates and screening advice, specifically for women who had dense breasts earlier but are now undergoing different diagnostic tests with possible changes in their breast density over time [8].

In a study, researchers found that patients screened with DBT were less likely to be assessed with high‐density breasts than those who underwent only digital mammography (OR = 0.69 and 0.43, respectively; *p* < 0.001). Similarly, synthetic mammography with DBT images was also less likely to be classified as high density in comparison to digital mammography with DBT (OR = 0.62; *p* < 0.001) [9]; these results show how breast density might be categorized differently even among breast radiographic modalities.

Another study compared the measurement of breast tissue density using full‐field digital mammography (FFDM), DBT, and MRI with semiautomated software, which has never been done before [10]. The study involved 48 patients who were all women with a mean age of 41 years, with an age range between 35 and 67 years old. It was noticed that there were strong positive linear correlations between the three imaging modalities concerning density measures: *r* = 0.95 (MRI and DBT), *r* = 0.97 (FFDM and DBT), and *r* = 0.87 (FFDM and MRI), with highly significant differences between FFDM and DBT/MRI (*p* < 0.0001) but no significant differences between DBT and MRI (*p* > 0.05). FFDM overestimated breast density compared to DBT and MRI by 15.1% and 16.2%, respectively. This indicates that incorporating automated and or structured methods could provide a more standardized and consistent approach to assessing breast density for women across breast imaging modalities. However, to the authors′ knowledge, the agreement or variations in radiologist assessment across all breast imaging and screening modalities have not been evaluated. Therefore, this study was aimed at evaluating the agreement between different imaging modalities (mammography, synthetic mammography of digital breast tomosynthesis [SM‐DBT], US, and MRI) in breast density assessment and determining which modality best predicts breast cancer risk.

## 2. Methods

Ethical approval was obtained from King Saud University Medical City Institutional Review Board (IRB) (Approval Project No. E‐23‐8043).

Data were retrospectively collected for women who underwent mammography, DBT, US, and breast MRI at King Khalid University Hospital (KKUH) between September 2018 and September 2023. Due to the study′s retrospective nature, a consent waiver was granted by the ethical committee. Additionally, all participant information was removed, and all images were anonymized.

This study included women aged 30 years and older with or without a diagnosis of breast cancer who underwent imaging before any surgical intervention or treatment for breast abnormalities. Women who had undergone breast implants, breast plastic surgery, prior mastectomy, or prior breast surgery were excluded. Because imaging referrals were based on clinical indication rather than routine screening, only cases with complete records for all three modalities were eligible, and women with incomplete imaging datasets were excluded.

All breast cancer cases included in this study were confirmed by histopathology results as malignant and were diagnosed within 3 months following the latest imaging examinations. Two breast imaging consultant radiologists, each with more than 10 years of experience, prospectively and independently assessed breast density for all modalities. The examinations were randomly divided between them, and each radiologist interpreted approximately half of the total cases. As the same examination was not read by both radiologists, no consensus or disagreement resolution process was required. Breast density was assessed using the American College of Radiology (ACR) Breast Imaging Reporting Data System (BI‐RADS) fifth edition breast composition categories; these are Category A (the breasts are almost entirely fatty), Category B (there are scattered areas of fibroglandular density), Category C (the breasts are heterogeneously dense), and Category D (the breasts are extremely dense) [11], in addition to the visual analog scale (VAS) to provide percentage density (%PD) [12] and assigned a BI‐RADS ACR fifth edition breast density category for each exam [11]. In parts of the analysis, women were categorized to be either dense or nondense, where Categories A and B were considered nondense, while women with Categories C and D were considered dense.

### 2.1. Statistical Analysis

Descriptive statistics such as frequencies and percentages were computed for categorical variables. The continuous variables VAS were presented in mean ± standard deviation (SD) and median (interquartile range [IQR]). The agreement between dense and nondense breasts was assessed using Cohen′s kappa level of agreement for categorical data, and the correlation between percentage breast densities %PD was determined using the Spearman correlation coefficient (*ρ*). Additionally, we evaluated the best predictor for breast cancer based on breast density using the odds ratios (ORs) of logistic regression for cancerous versus noncancerous cases. The Statistical Package for the Social Sciences Version 27.0 (SPSS Inc., Chicago, Illinois, United States) was used for statistical analysis, and a *p* value < 0.05 was considered significant.

## 3. Results

A total of 77 women were included in the study; the mean age was 47.34 years (SD = 8.66), with the majority (32, 41.6%) falling in the 41–50‐year age group. These women were selected from approximately 1000 breast MRI examinations performed during the study period. Breast MRI, DBT/mammography, and US are not routinely recommended simultaneously for all women, and clinical referrals for the full set of modalities are based on individual presentation, prior findings, and radiologist recommendations. Therefore, not all screened women underwent all three modalities. For the purpose of this study, only women who had complete records for mammography/DBT, US, and MRI within the same clinical episode were included. Women with missing data or without complete imaging were excluded, which accounts for the final sample size of 77 and reflects the retrospective nature of the study. More than two‐thirds of this sample were noncancer cases (67.5%), while the remainder were diagnosed with confirmed breast cancer. Most of the women (58.4%) had symptoms at the time of imaging, as seen in Table 1. Approximately 29% were postmenopausal, around 86% of the women had one or more children, and 31 women reported having a family history of breast cancer. A detailed description of the sample characteristics is provided in Table 1.

**Table 1:** Demographic characteristics of the study participants, *n* = 77.

**Characteristics**	**Frequency (** **n** **)**	**Percentages (%)**
Age in years		
Mean ± SD	47.34 ± 8.66	
Median (IQR)	47 (12)	
BMI (kg/m^2^)		
Mean ± SD	33.66 ± 7.29	
Median (IQR)	30.81 (10.93)	
Cancer status		
Cancerous	25	32.5
Noncancerous	52	67.5
Symptoms		
Yes	45	58.4
No	32	41.6
Number of children		
None	11	14.3
1–4 children	33	42.9
> 5 children	24	31.2
Unknown	9	11.7
Menopause		
Yes	22	28.6
No	43	55.8
Unknown	12	15.6
Family history of breast cancer		
Yes	31	40.3
No	40	51.9
Unknown	6	7.8

### 3.1. Breast Density Distribution Across Imaging Modalities

**Figure 1:**
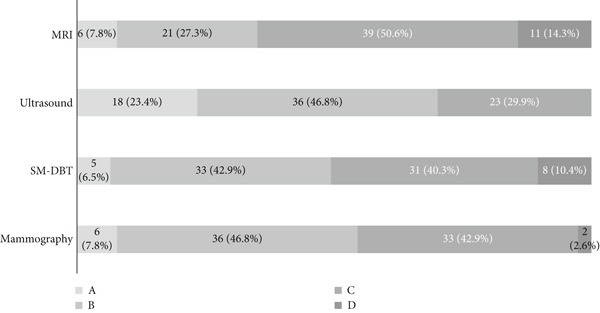
Breast density distribution across Breast Imaging Reporting and Data System (BI‐RADS) categories (A, B, C, and D) for mammography, DBT (digital breast tomosynthesis), MRI (magnetic resonance imaging), and ultrasound. For ultrasound assessment, Category D was not included in accordance with the American College of Radiology ACR BI‐RADS fifth edition.

In this study, radiologists prospectively categorized breast density using the ACR BI‐RADS breast composition categories, fifth edition, for mammography, SM‐DBT, US, and breast MRI. Figure 1 shows the different distribution of categories among breast imaging modalities. The number of women categorized with breasts that are almost entirely fatty (Category A) was similar for mammography and MRI, *n* = 6 (7.8%), and almost similar to SM‐DBT (*n* = 5). Using US images, on the other hand, radiologists categorized 18 women to be with entirely fatty breast tissue (as shown in Figure 1).

Regarding categorizing women with extremely dense breasts (Category D), US did not include this category as per the ACR guidelines. Mammography had the lowest number of women in the D category, 2.6%, while MRI had the highest number of women categorized with extremely dense breasts. By combining the two nondense categories (A and B) and dense categories (C and D), or C alone for US, MRI showed the highest proportion of dense breasts (64.9%), followed by DBT (50.6%), mammography (45.5%), and US (29.9%) (Figure 1).

**Figure 2:**
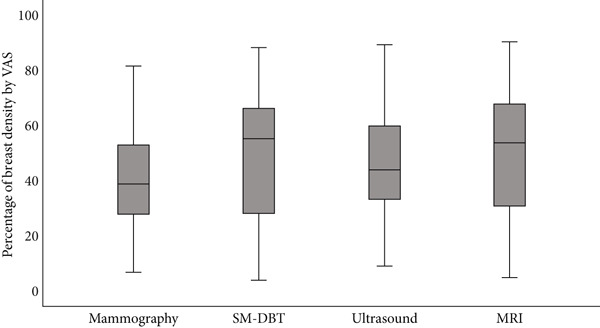
Boxplot showing the distribution of VAS breast density percentage across four imaging modalities: mammography, digital breast tomosynthesis (DBT), ultrasound, and MRI. The gray boxes represent the interquartile range (IQR), and the black lines indicate the median percentage for each modality. This visualization highlights the variation in breast density assessments across different imaging modalities.

Using continuous VAS, radiologists assessed the PD% of breast images across all modalities. The mean VAS was highest for MRI (50.01*%* ± 23.30*%*), followed closely by DBT (49.34*%* ± 23.28*%*) and US (44.99*%* ± 21.41*%*). Mammography, on the other hand, showed slightly lower values (40.33*%* ± 17.87*%*). The range of VAS was wide across all modalities, ranging from 6.75% to 81.75% for mammography and 5.00%–90.50% for MRI (Table 2). The distribution of %PD values for each modality is visually seen in Figure 2. The boxplots show that MRI and DBT assessments have higher values, while mammography has a narrower range.

**Table 2:** Breast density percentage of the same women (*n* = 77) using VAS across four modalities.

	**Mean**	**SD**	**Median**	**IQR**	**Min**	**Max**
Mammography	40.33	17.87	39.25	25	6.75	81.75
SM‐DBT	49.34	23.28	55.5	38	4	88.5
Ultrasound	44.99	21.41	44	26.5	9	89.5
MRI	50.01	23.30	54	37	5	90.5

### 3.2. Agreement Between Breast Density Assessment Across Breast Imaging Modalities

The findings from the correlation analysis of radiologists′ categorization of breast density across modalities revealed good agreement between mammography and SM‐DBT, with a Cohen′s kappa (*k*) of 0.535 when categorizing women as dense or nondense. There was moderate agreement between mammography and MRI (*k* = 0.452) and poor agreement between mammography and US (*k* = 0.373). The results also demonstrated moderate agreement between SM‐DBT and MRI (*k* = 0.472) and between SM‐DBT and US (*k* = 0.417). The lowest agreement was found between US and MRI (*k* = 0.360). The differences between methods were significant, *p* < 0.05, except between mammography and SM‐DBT (Table 3).

**Table 3:** Cohen′s kappa correlation analysis between modalities (ACR BI‐RADS categories fifth edition combined) (dense and nondense).

**Analysis between modalities**	**Kappa value**	**p** **value** ^∗^
Mammography vs. SM‐DBT	0.535	0.48
Mammography vs. ultrasound	0.373	0.023
Mammography vs. MRI	0.452	0.0026
SM‐DBT vs. ultrasound	0.417	0.0015
SM‐DBT vs. MRI	0.472	0.027
Ultrasound vs. MRI	0.360	<0.001

^∗^McNemar′s tests for paired comparisons between imaging modalities.

Similar to the categorical breast density assessment, the correlation analysis of %PD assessments by radiologists using VAS across all imaging modalities also revealed varying levels of agreement. Moderate positive correlations were observed between mammography and SM‐DBT (*ρ* = 0.49), mammography and MRI (*ρ* = 0.56), and SM‐DBT and MRI (*ρ* = 0.56), indicating more consistent assessments among these modalities. In contrast, US showed weaker correlations with the other methods, with the highest being between SM‐DBT and US (*ρ* = 0.42), while the correlation between mammography and US was particularly low (*ρ* = 0.08). Table 4 presents these correlations.

**Table 4:** Correlation between percentage breast densities of mammography, DBT, ultrasound, and MRI.

**VAS correlation** ^∗^	**Mammography**	**SM-DBT**	**Ultrasound**	**MRI**
Mammography	1.00			
SM‐DBT	0.492 ^∗∗^	1.00		
Ultrasound	0.082	0.421 ^∗∗^	1.00	
MRI	0.558 ^∗∗^	0.562 ^∗∗^	0.287 ^∗∗^	1.00

^∗^Spearman′s rank correlation.

^∗∗^Correlations are statistically significant, *p* < 0.05.

### 3.3. Comparison of Breast Cancer Risk Prediction of Imaging Modalities Based on Breast Density

The OR analysis for breast cancer prediction based on dense versus nondense classifications across four imaging modalities—mammography, DBT, US, and MRI—revealed significant associations between mammography and MRI. Women classified as dense by mammography had 3.09 times higher odds of having breast cancer compared to nondense women (OR = 3.09, 95% CI: 1.14–8.33, *p* = 0.026). Similarly, MRI showed a strong association, with dense women having 4.16 times higher odds of cancer (OR = 4.16, 95% CI: 1.25–13.84, *p* = 0.020). In contrast, DBT (OR = 2.24, *p* = 0.108) and US (OR = 2.00, *p* = 0.182) were not statistically significant (as shown in Table 5).

**Table 5:** Risk of breast cancer for dense versus nondense for each imaging modality.

**Variables**	**Dense**		**Nondense**		**OR**	**95% CI**	**p** **value**
	**Cancer**	**Noncancer**	**Cancer**	**Noncancer**			
Mammography	16	19	9	33	3.09	1.14–8.33	0.026
SM‐DBT	16	23	9	29	2.24	0.84–5.99	0.108
Ultrasound	10	13	15	39	2.00	0.72–5.53	0.182
MRI	21	29	4	23	4.16	1.25–13.84	0.020

## 4. Discussion

Breast density is a significant factor in breast cancer screening and diagnosis [2, 7, 13]. The dense breast tissue appears white on mammograms, as do tumors, making it difficult to detect cancer [14]. Women with dense breasts not only have a higher risk of breast cancer but also face decreased sensitivity of mammography, leading to potential delays in diagnosis [1, 2, 15]. Thus, accurate breast density assessment is critical for effective breast cancer screening and risk prediction [7]. Radiologists mostly assess breast density using mammography images. However, other imaging modalities such as DBT, US, and MRI are also employed in screening, especially due to increased breast density [16] or when tracking breast tissue changes over time to assess cancer risk [17].

Our findings highlight discrepancies in breast density categorization across the different imaging modalities for the same sample of women. Mammography showed a lower proportion of women with dense breasts (45.5%) compared to MRI (64.9%) and SM‐DBT (50.6%) but higher than US (29.9%).

Regarding the correlation between methods in categorical breast density assessments (BI‐RADS), our results showed that radiologists had a moderate agreement of breast density when comparing mammography to SM‐DBT (*k* = 0.535) compared to other methods. This is likely due to the similarity in these imaging techniques in terms of how they depict the fibroglandular tissue of the breast in the radiographic image. MRI had slightly less agreement with mammography (*k* = 0.452); nevertheless, MRI identified more women with dense breast tissue. This is important because MRI is now recommended for screening women with extremely dense breasts [16]; thus, relying on breast density assessment using MRI could be more appropriate for monitoring breast density for those women.

The continuous scale correlation analysis using %PD assessments revealed moderate positive correlations between mammography and SM‐DBT (*ρ* = 0.49). Additionally, the correlation between SM‐DBT and MRI (*ρ* = 0.56) was identical to that between mammography and MRI. This indicates that, similar to the categorical assessments, mammography and SM‐DBT show good agreement, while MRI is correlated more strongly with mammography methods than US.

Nevertheless, the observed variations in breast density assessment are attributed to the inherent differences in imaging techniques. Mammography, a 2D imaging modality, often struggles with overlapping tissue, which can obscure dense areas. In contrast, DBT, a 3D imaging modality, provides better tissue delineation, reducing the impact of overlapping structures. MRI, known for its high contrast resolution, excels in differentiating between dense and nondense tissue, explaining the higher density categorization as observed in this study. US, while beneficial for supplemental screening, particularly in dense breasts, is operator‐dependent and less consistent in density categorization [18].

This variability between modalities seen in this study is consistent with previous studies. For instance, Tagliafico et al. in 2013 investigated the measurement of breast tissue density using FFDM, DBT, and MRI with semiautomated software [10]. Their findings revealed differences in percentage breast density between FFDM and DBT and between FFDM and MRI (*p* = 0.0001). Similarly, another study examined the variation between mammography and DBT in BI‐RADS breast density assessments [9]. The study indicated that women screened with DBT were less likely to be categorized as having high‐density breasts compared to digital mammography (OR = 0.69 and 0.43, respectively; *p* < 0.001). Additionally, synthetic mammography with DBT also showed a lower likelihood of high‐density categorization compared to digital mammography with DBT (OR = 0.62; *p* < 0.001). This variability in breast density categorization across different imaging modalities has profound implications for clinical decision‐making and screening protocols, and inconsistent breast density assessments can lead to varied risk stratification and, consequently, different screening recommendations [2].

Moreover, this study provided a comparison of breast cancer risk prediction across different imaging modalities based on breast density classification, aiming to provide valuable insights into the role of breast density as a risk factor for breast cancer and the reliability of each modality in identifying women at higher risk. The results demonstrated that mammography and MRI are the most reliable imaging modalities for predicting breast cancer risk based on breast density classification.

Among the evaluated 77 women, those classified as having dense breasts on mammography had higher odds of a breast cancer diagnosis than those with nondense breasts (OR = 3.09, 95% CI: 1.14–8.33, *p* = 0.026). This observation is in line with prior reports that link increased mammographic density to a greater likelihood of cancer detection on mammography [19–21].

Similarly, MRI data in this study indicated higher odds for breast cancer among women with dense breasts (OR = 4.16, 95% CI: 1.25–13.84, *p* = 0.020). MRI′s known sensitivity to dense breast tissue and its ability to provide detailed contrast between normal and abnormal tissues may explain this observed association [16].

DBT and US did not show statistically significant associations between density classification and cancer in this cohort (*p* > 0.05). These findings may reflect limited statistical power, modality‐specific sensitivity differences, or heterogeneity within the sample rather than a true absence of association. Because only 77 of the approximately 1000 available patients were included, the results are subject to potential selection and verification biases and should not be generalized to the broader population. Accordingly, the findings are presented as descriptive and hypothesis‐generating rather than confirmatory.

Several limitations should be acknowledged. The small sample size reduced the statistical power of the analysis and limits the generalizability of the findings. The limited number of cancer cases also restricts the ability to draw firm conclusions about the predictive role of breast density in cancer risk. Moreover, the cross‐sectional design precludes any assessment of temporal or causal relationships, and potential confounders such as hormonal status or lifestyle factors were not fully accounted for.

Despite these limitations, this study provides valuable preliminary insights into the level of agreement among different breast imaging modalities in assessing breast density and their potential implications for cancer risk evaluation. Larger, multicenter, and prospectively designed studies are warranted to validate and expand upon these findings.

## 5. Conclusion

This study provides the first direct comparison of breast density assessment across all standard breast imaging modalities based on radiologist evaluation. The findings revealed considerable variability in breast density assessment between modalities, with MRI showing greater sensitivity in identifying women with dense breasts and demonstrating potential utility in predicting breast cancer risk. These results suggest that MRI may serve as a valuable adjunct technique for women with dense breasts, potentially enhancing early cancer detection. The study also highlights the importance of developing standardized protocols and personalized imaging strategies in clinical practice. Future research, particularly larger multicenter studies and investigations incorporating artificial intelligence and automated breast density measurement across modalities, is warranted to build upon these preliminary findings.

**Nomenclature**
SM: synthetic mammography
DBT: digital breast tomosynthesis
US: ultrasound
MRI: magnetic resonance imaging
ACR: American College of Radiology
VAS: visual analog scale
%PD: percentage density
OR: odds ratio
SD: standard deviation
FFDM: full‐field digital mammography
IQR: interquartile range

## Conflicts of Interest

The authors declare no conflicts of interest.

## Funding

The authors have nothing to report.

## Data Availability

The data that support the findings of this study are available from the corresponding author upon reasonable request.
